# Supplementary material for: The Role of ROR1 in Chemoresistance and EMT in Endometrial Cancer Cells
Source: Medicina (Kaunas). 2023 May 21;59(5):994. doi: 10.3390/medicina59050994 (PMC10223135; doi:10.3390/medicina59050994)
Supplement: Supplementary file 1 [file medicina-59-00994-s001.zip › Supplementary Figures.pdf]

Figure S1. (a) Original western blot replicates and (b) densitometry readings/intensity ratio of each band for Figure 1A

(a)

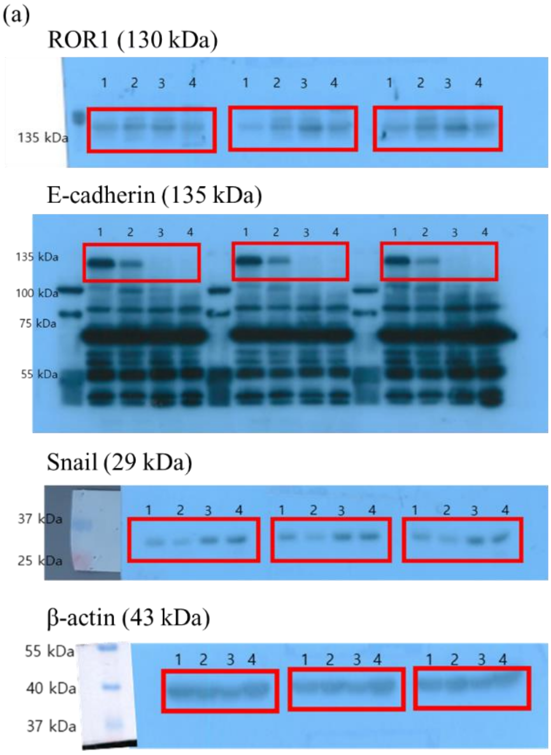

Rane 1, Ishikawa; 2, HEC-1; 3, SNU-539; 4, SNU-685

(b)

cell line	ROR1	E-cadherin	Snail
Ishikawa	0.139378159	0.843739912	0.55315189
	0.242448459	0.867375895	0.321935187
	0.35728721	0.848327757	0.777582222
HEC-1	0.447942689	0.421157375	0.804191371
	0.499488885	0.420160045	0.611225555
	0.510584109	0.422964412	0.361926969
SNU-539	1.137802983	0	0.911177797
	1.098957042	0	0.759589986
	1.316275015	0	0.746498212
SNU-685	0.397301608	0	0.398367891
	0.355074173	0	0.726203842
	0.220760006	0	0.835694044

Figure S2. (a) Original western blot replicates and (b) densitometry readings/intensity ratio of each band for Figure 3A

(a)

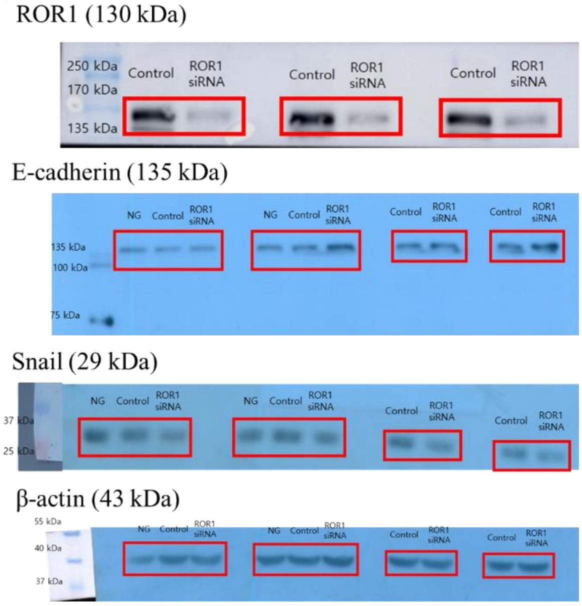

(b)

	ROR1	E-cadherin	Snail
control	0.810450067	0.710302826	0.798264951
	0.825846953	1.089789884	0.746976591
	0.886789513	1.133686653	0.653572422
ROR1 siRNA	0.104979761	1.602574315	0.52979098
	0.080524016	1.527009242	0.476053498
	0.093489877	1.925215431	0.292810667

Figure S3. (a) Original western blot replicates and (b) densitometry readings/intensity ratio of each band for Figure 3B

(a)

ROR1 (130 kDa)

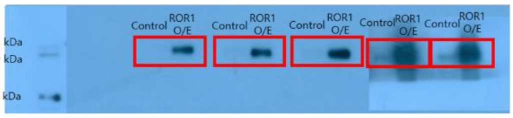

E-cadherin (135 kDa)

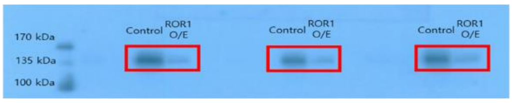

Snail (29 kDa)

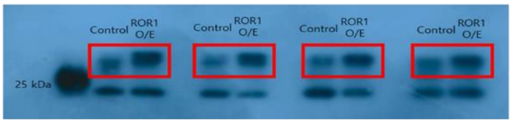

β-actin (43 kDa)

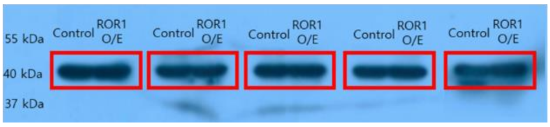

(b)

	ROR1	E-cadherin	Snail
control	0.236401991	1.126788316	0.518080912
	0.094069538	0.96776859	0.555911342
	0.184821376	0.995587519	0.461335739
ROR1 O/E	1.314115015	0.44699226	1.250669653
	1.512173138	0.457730128	1.513693667
	1.646261382	0.549946693	1.341844266

Figure S4. (a) Original western blot replicates and (b) densitometry readings/intensity ratio of each band for Figure 3C and 3D.

(a)

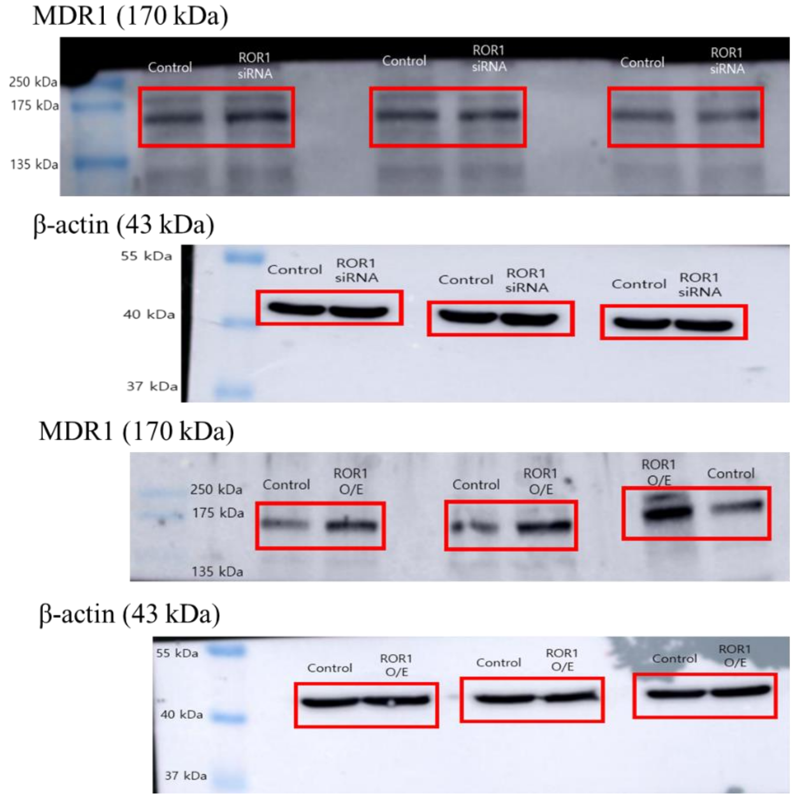

(b)

	**MDR1**
control	1.058212448
	0.689348673
	1.038818761
ROR1 siRNA	1.245649951
	0.825100804
	0.659673422

	**MDR1**
control	0.888714519
	0.632756008
	0.670140509
ROR1 O/E	1.305213261
	1.338373342
	1.340939445

Figure S5. Expression of ROR1 and EMT markers in Ishikawa cell line and Ishikawa^{Taxol} cells and investigation of anticancer drug resistance

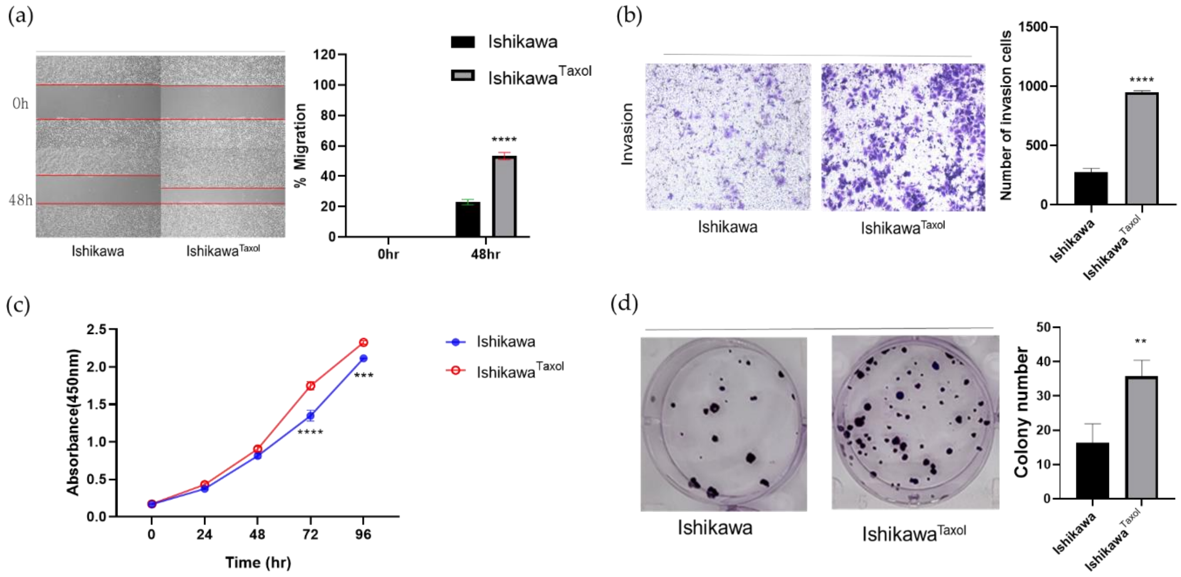

Figure S6. (a) Original western blot replicates and (b) densitometry readings/intensity ratio of each band for Figure 4A.

(a)

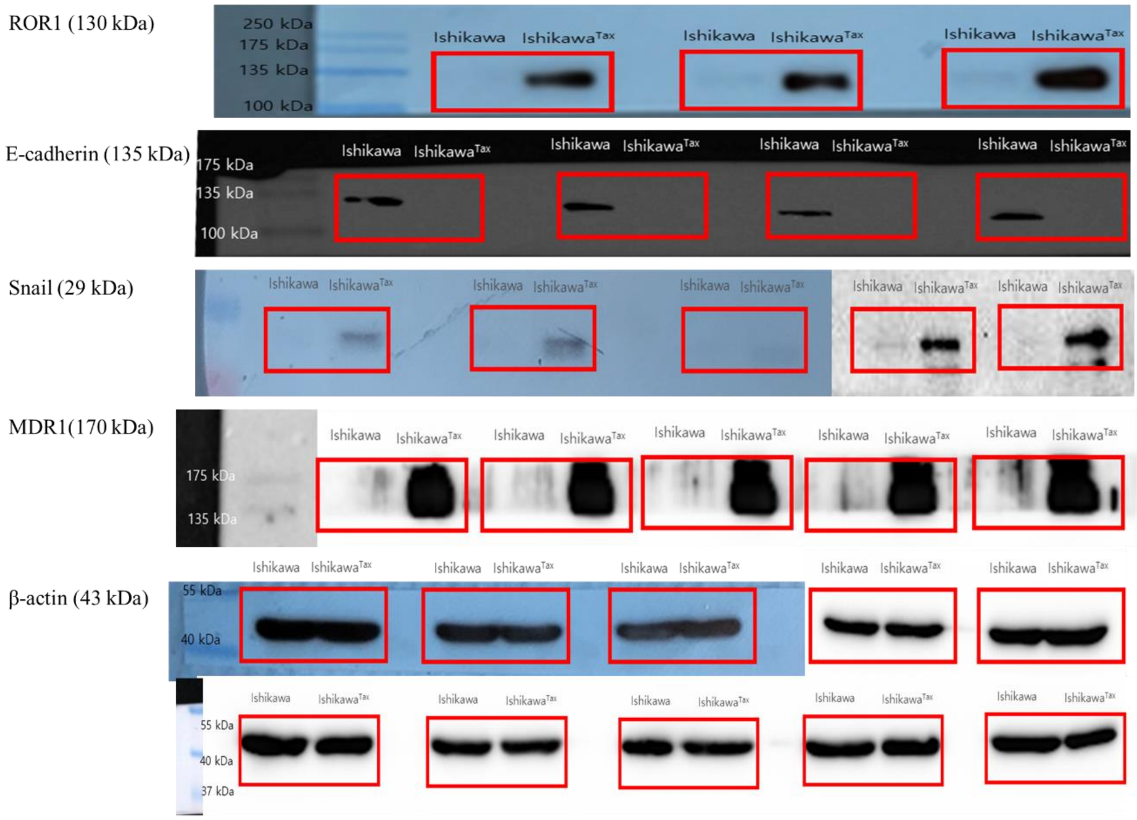

(b)

cell line	ROR1	E-cadherin	Snail	MDR1
Ishikawa	0.18916234	0.936288317	0.191745051	0.147139925
	0.195426602	0.801147198	0.2245788	0.120616066
	0.190309256	0.792611973	0.184296787	0.121231419
Ishikawa^{Taxol}	0.806494503	0	0.691445367	1.112617349
	0.921446853	0	0.65883584	1.227735711
	0.792062027	0	0.888006589	1.045984164
