# Supplementary material for: The Role of ROR1 in Chemoresistance and EMT in Endometrial Cancer Cells
Source: Medicina (Kaunas). 2023 May 21;59(5):994. doi: 10.3390/medicina59050994 (PMC10223135; doi:10.3390/medicina59050994)
Supplement: Supplementary file 1 [file medicina-59-00994-s001.zip › Supplementary Table S1.pdf]

Table S1. The accession number, database name and the genetic information with STR profile of each cell lines

Cell name	Ishikawa	HEC-1	SNU-539	SNU-685
Accession number	99040201	HTB-113	00539	00685
Database name	ECACC General Collection (ECACC)	American Type Culture Collection (ATCC)	Korean Cell Line Bank (KCLB)	Korean Cell Line Bank (KCLB)
Genetic information (STR profile)	Amelogenin: X<br>CSF1PO: 11,12<br>D13S317: 9,12<br>D16S539: 9<br>D5S818: 10,11<br>D7S820: 9,10<br>THO1: 9,10<br>TPOX: 8<br>vWA: 14,17<br>D7S820: 8,12	Amelogenin: X<br>CSF1PO: 10,12<br>D13S317: 11,16<br>D16S539: 11,12<br>D5S818: 11,13<br>D7S820: 9,11<br>THO1: 6,7<br>TPOX: 8,11<br>vWA: 18	D3S1358: 15,18<br>vWA: 15,17<br>FGA: 23,26<br>Amelogenin: X<br>TH01: 6,9<br>TPOX: 8,11<br>CSF1PO: 11,12<br>D5S818: 11<br>D13S317: 14<br>D7S820: 8	D3S1358: 14<br>FGA: 21<br>Amelogenin: X<br>TH01: 7<br>TPOX: 9<br>CSF1PO: 11<br>D5S818: 12<br>D13S317: 8<br>D7S820: 8,12
